# Cultural variability in dementia caregiver motivations: Unraveling unique and common drivers

**DOI:** 10.1177/14713012251327461

**Published:** 2025-04-14

**Authors:** Najoua Lazaar, Floor Flurij, Rozemarijn L van Bruchem-Visser, Janne M Papma, Sanne Franzen

**Affiliations:** Department of Neurology, 6993 Erasmus MC University Medical Center Rotterdam, Netherlands; Department of Internal Medicine, Erasmus MC University Medical Center Rotterdam, Netherlands; Department of Neurology, Erasmus MC University Medical Center Rotterdam, Netherlands; Department of Internal Medicine, Erasmus MC University Medical Center Rotterdam, Netherlands; Department of Neurology, Erasmus MC University Medical Center Rotterdam, Netherlands

**Keywords:** caregiver burden, cultural diversity, cultural competency, dementia, caregivers

## Abstract

**Introduction:** In the Netherlands, approximately 800,000 individuals act as informal caregivers for people with dementia. Current policies prioritize care within the home setting, often relying on informal caregivers for support, assisted by care professionals. Given the wide ethnocultural diversity among dementia caregivers in the Netherlands, it is crucial to understand how these cultural differences influence caregiving. Given the emphasis on researching barriers to providing care in literature, this study specifically focused on motivators and facilitators to providing care. **Methods:** We conducted semi-structured interviews, both with caregivers of native Dutch patients (*n* = 11) and caregivers of patients with a migration background (*n* = 9), who all provided care at home for patients with dementia. Data was collected using an interview guide and open, axial and selective coding were used to analyze the transcripts of the interviews in Atlas.ti. **Results:** Four themes were identified. First, culturally shared motivators to providing care emerged, such as having a unique bond with the person with dementia and themes of reciprocity. Second, culture specific perspectives were identified, with Dutch caregivers often spontaneously reporting considering professional care, while culturally diverse caregivers stressed the available network of informal caregivers and their ability to persevere. Third, personal philosophies on life were a driver to provide care for caregivers of native Dutch patients, while religion mainly served as a source of strength in continuing to provide care in the culturally diverse group. Lastly, adult child caregivers benefit from supportive home environments and from using structure and routine in providing care. **Discussion:** Our findings show that the decision to provide care often seems driven by reciprocity and the prior quality of the relationship with the person with dementia, contrasting with previous work suggesting that religion is a main reason to provide care. Several recommendations are made how care professionals can take these factors into consideration when assisting caregivers.

## Introduction

Dutch policies aim to encourage older adults to ‘age in place’ (Rijksoverheid, 2022), emphasizing the family’s essential role in providing care at home for individuals with dementia. Healthcare professionals offer support to informal caregivers in this process. There are over 800,000 individuals in the Netherlands who provide some form of care to a person with dementia (Sociaal Cultureel Planbureau, 2020). There is a growing number of caregivers from culturally diverse backgrounds; this is linked to the 3-4x higher prevalence of dementia in some diverse demographic groups in the Netherlands than in native Dutch (Parlevliet et al., 2016). This higher prevalence has in turn been associated with a higher prevalence of risk factors for dementia such as diabetes, hypertension, cardiovascular diseases, and low levels of education (Parlevliet et al., 2016). In sum, rapidly growing numbers of patients with dementia are expected among diverse populations in the Netherlands, resulting in increasing numbers of informal caregivers with a migration background. These caregivers may experience particularly high levels of burden as some demographic groups tend to utilize professional care less frequently and rely more on informal dementia support (Duran-Kirac et al., 2022). This has important implications for the provision and organization of dementia care (for more context see: text box 1).

**Text box 1. Research in context: Aging in the Netherlands and core values of the Dutch health care system**

On January 1, 2024, the Netherlands had 3,677,228 residents aged 65 or older, totaling 20.5 percent of the population. This marks a significant increase in the aging population from 12.9 percent in 1990 (Central Bureau of Statistics, 2024). Currently, an estimated 300,000 people are living with dementia in the Netherlands (Alzheimer Nederland, 2024; Central Bureau of Statistics, 2024), who are cared for by 800,000 informal caregivers (Sociaal Cultureel Planbureau, 2020). Dutch policies encourage older adults to “*age in place*,” emphasizing the essential role of families in providing at-home care for individuals with dementia (Rijksoverheid, 2022). Healthcare professionals support informal caregivers in this process. Although the national care standards for dementia (Zorgstandaard Dementie, 2020) place significant emphasis on cultural diversity and potentially differing needs and wishes of patients with a migration background, the underlying values of the Dutch healthcare system may not reflect the values prioritized by all patients. Medical ethics training in the Netherlands primarily emphasizes the four principles of autonomy, beneficence, non-maleficence, and justice (Bolt et al., 2010; Ten Have et al., 2009), with an increasing focus, however, on patient autonomy (e.g. in the Dutch Doctors Federation’s guidelines (KNMG, 2024). Furthermore, in 2020, shared decision making was added as a prerequisite in doctor-patient encounters in Dutch law (Van Der Weijden et al., 2022). Autonomy and shared decision-making in patient-doctor interactions may not necessarily align with the preferences of individuals who score high on relational interdependence holding strong beliefs in social hierarchy, who may prefer involving family members in medical decision-making (Scherr et al., 2022).

Informal caregivers (e.g. children or partner) of culturally diverse patients with dementia experience high levels of caregiver burden, particularly with increasing dementia severity or in cases where the caregiver is the spouse (Franzen et al., 2021). Burden levels can be positively influenced by the possibility to mobilize help within the network of the caregiver, such as having a social network to fall back on or by using formal care services (Ahmad et al., 2022). Gender norms within the care context also influence the caregiver’s experience and level of experienced burden (Ahmad et al., 2022). Research suggests that in diverse families, caregiving responsibilities are often unequally distributed, with one specific family member – typically a woman – bearing the primary caregiving burden without relying on professional care services (Ahmad et al., 2022; Van Wezel et al., 2018). Several factors may impact the caregiving experience, such as a family’s and individual’s positions on the collectivistic-individualistic continuum. In collectivistic cultures, the family plays an essential role; important principles are showing respect for older family members and taking responsibility for one another by organizing care together (Shibusawa, 2024). A seminal work by Markus and Kitayama (1991) highlights that many North American and European cultures prioritize an independent self-concept, viewing the self as separate, autonomous, and defined by individual traits. In contrast, people from many Asian, African, and Latin American cultures typically embrace an interdependent self-concept, seeing themselves as intrinsically connected to a larger social network, including family and others with whom they share bonds (Knight et al., 2022).

Another potentially relevant cultural factor is the continuum of femininity-masculinity. Historically, caregiving has been considered as a feminine activity (Miller et al., 2024). Some studies have considered caregiving through a masculine lens wherein caregiving responsibilities are often delineated in terms of more functional tasks (Poisson et al., 2023). Interestingly, Nielsen, Nielsen, and Waldemar (2021) found that hierarchical and gendered roles and expectations were increasingly being challenged and negotiated in minority ethnic families. Last, cultural perspectives may also impact caregiver experiences. Caregivers from diverse backgrounds that do not personally provide care to their loved one may experience feelings of shame, which in turn serves as a barrier to turn to professional services (Alzheimer Europe, 2020). In the case of religious caregivers, particularly within the Muslim community, other members may interpret accepting help from outside the community as rejecting Allah’s (God’s) test or divine will (Alzheimer Europe, 2020; Van Wezel et al., 2018).

Caregivers of diverse individuals with dementia may encounter several barriers in their role of informal caregiver. First, they may experience difficulties in adjusting their lives and have limited knowledge or awareness of available care services (Alzheimer Europe, 2018; Duran-Kirac et al., 2022). Second, access to and use of dementia-related healthcare may subsequently be influenced by the ‘approachability’, which encompasses transparency and the provision of information regarding available dementia services and treatments, and ‘acceptability’, referring to the cultural and social factors that may affect the utilization of these services (Duran-Kirac et al., 2022). Furthermore, seeking assistance or support may be particularly challenging when a lack of trust further undermines the ‘approachability’ and ‘acceptability’ of the available dementia services (Duran-Kirac et al., 2022). Previous encounters with discrimination and prejudice can impact the ability to effectively navigate healthcare systems and services (Alexander et al., 2022). Lastly, dementia is often diagnosed at a later stage in diverse populations (Goudsmit et al., 2022), potentially impacting access to resources and services.

As is apparent from this summary of the existing literature, there has been an emphasis on factors that stand in the way of good care, while limited attention is paid to what motivates and enables (i.e. facilitates) caregivers (to continue) to provide care in the home setting. Ahmad et al. (2020) did show that factors such as love and religion can be motivators to provide care.

Given the policy emphasis that is placed on ‘aging in place’, it is vital to examine these motivators and potential facilitators to providing (often long-term) care in the home setting, as these policies are likely attained only by both overcoming barriers to care and investing in facilitating factors. The goal of this study is therefore to examine the variation in culturally diverse informal caregivers’ motivators and facilitators to providing care. The following research question was formulated: *What facilitators can be identified to enhance the provision of long-term care at home by culturally diverse informal caregivers?* By examining these factors, the study seeks to identify ways to lift existing barriers to care and to integrate facilitators into care practices and policies, thereby increasing the likelihood of successful aging in place with optimal quality of life.

## Methods

### Recruitment of participants

We enrolled informal caregivers of patients with a wide variety of dementia subtypes and a broad range of time since the diagnosis (3 weeks up to 10 years prior). The participants were mainly recruited through the Alzheimer Center of the Erasmus Medical Center and its multicultural memory clinic in Rotterdam, the Netherlands. The attending medical professional asked the caregiver during the visit whether they would consider participating in the study, with the first author following up with detailed study information and obtaining informed consent. Two caregivers were included while the patient was temporarily admitted to the inpatient ward of the department of Geriatric medicine of the Erasmus Medical Center in Rotterdam. A subset of caregivers was included using snowball sampling, i.e., included caregivers suggested possible other participants for the study. The caregivers were interviewed between January 2022 and June 2023.

In selecting participants, the study explicitly aimed to capture intersecting dimensions of diversity, particularly regarding the cultural backgrounds of both patients and caregivers. Accordingly, the inclusion criteria were broadly formulated: participants were eligible if they were informal caregivers (e.g. family or friends) of patients with a formal dementia diagnosis, including a wide range of dementia subtypes and disease stages. Additionally, caregivers were required to provide care within the home environment of the person with dementia, though they were not required to cohabitate. We excluded professional caregivers and caregivers of patients not formally diagnosed. Although not a strict criterion, our aim was to recruit a balanced sample of caregivers with and without a migration background, representative of the demographics of major cities in the Netherlands (Central Bureau of Statistics, 2022). Additionally, all caregivers needed to be 18 years or older.

### Data collection

The data for this study were collected by conducting semi-structured, in-depth interviews and analyzing the transcripts using thematic analysis in a population of dementia caregivers of both native Dutch patients and patients with migration backgrounds. To ensure rigor and trustworthiness in the research process, several strategies were employed, including member checking to validate findings with participants, and researcher triangulation to validate the data. The data were collected by the first author (NL) through interviews that were conducted face-to-face (*n* = 14), online via Teams (*n* = 4) or through telephone (*n* = 2). The average duration of the interviews was 45 minutes. All participants spoke Dutch during the interviews. The interviews were structured using an interview guide in Dutch (see the supplemental material for an English translation). The interview guide was developed partially based on existing literature, ensuring that key themes relevant to the experiences of the caregivers were included. This literature-informed approach helped to enhance the comprehensiveness and relevance of the questions. We inquired about for example the relationship between the caregiver and person with dementia prior to the diagnosis, but also asked questions to obtain a better understanding of the context of providing care, such as “*can you describe a day in your lives*?“, “*why do you provide care?*“, “*what are you proud of in taking care of your loved one*” and “*what helps you cope?*“. Furthermore, we collected information about the demographics of the caregiver and patient, dementia severity of the patient at the moment of the interview, and prior experiences with caregiving in general.

### Data analysis

The audio recordings were transcribed verbatim and analyzed independently by two authors (NL and FF) using Atlas.ti. We acknowledge that the potential impact of the researchers’ cultural backgrounds hold significance, particularly within the context of this study where the interplay between the different cultural contexts is salient. NL is primarily socialized within the Moroccan and Dutch culture; FF is mainly socialized within the Dutch culture (as are coauthors JMP, SF, and RvB). The transcripts of the interviews were analyzed by following the open, axial, and selective coding process (Babbie, 2020). In the open coding process, the researchers assigned labels to text elements of the transcripts. During axial coding the labels in the open coding process were examined and some of them were grouped in one overarching code. The overarching codes were then discussed between the researchers. The last step was the selective coding process where associations and connections were made to identify themes.

### Participant characteristics

Demographic data were collected for all participants (see Table 1). The sample consisted of patients born in different countries (with presumably different cultural backgrounds): the Netherlands, Belgium, Turkey, Morocco, China, and Suriname. In this study, interviews were conducted with 11 caregivers of native Dutch patients and 9 caregivers of patients with a migration background, with the aim of capturing a broad range of experiences and perspectives. The average age of the participants was 55 years (range 40–73 years) and the sample consisted of both male (*n* = 6) and female (*n* = 14) caregivers. Half of the participants were spouses (*n* = 10), eight were adult children, one participant cared for her father-in-law, and one person identified as the patient’s friend. In the case of the culturally diverse patients, caregivers were often adult children, while the caregivers of Dutch patients were often spouses. Half of the participants (*n* = 10) cohabited with the person with dementia. Most of the participants had no prior experience with caregiving nor had worked as a healthcare professional. Most patients were diagnosed with Alzheimer Disease Dementia (*n* = 14), but caregivers for patients with Frontotemporal Dementia (*n* = 3), Primary Progressive Aphasia (*n* = 1), Vascular Dementia (*n* = 1) and Mixed Dementia (Alzheimer’s Disease and Vascular Dementia, *n* = 1) are also represented in this sample. Caregivers assisted in instrumental activities of daily living (iADL), such as in finances, but some also provided extensive help with most activities of daily living (ADL), such as washing and getting dressed. As the interviews progressed, recurring themes related to caregiving experiences, challenges, and coping strategies became evident across both caregiver groups, signaling that key aspects of the research questions had been sufficiently addressed. At this point, the decision was made to halt further data collection, in line with the principle of data saturation.

**Table 1.** Characteristics of caregivers and patients.

#	Relationship to patient	Age	Sex^a^	Field of occupation of the caregiver	Type of education^b^	Country of origin patient	Diagnosis patient^e^	Time since diagnosis
1	Spouse	48	M	Nursing	Practical	Netherlands	PPA	Months
2	Spouse	59	F	Sales	Theoretical	Belgium	FTD	Three weeks
3	Daughter in law	55	F	IT / Tech	Theoretical	China	AD	Months
4	Spouse	73	F	Education	Theoretical	Netherlands	AD	Seven years
5	Spouse	66	M	Sales	Practical	Netherlands	FTD	One year
6	Spouse	71	M	Military	Theoretical	Netherlands	AD	Months
7	Daughter	40	F	Long term informal caregiver	Practical	Turkey	Mixed AD/VaD	One year
8	Spouse	59	F	Stay-at-home parent	Practical	Netherlands	AD	Weeks
9	Spouse	60	F	Manufacturing	Practical	Netherlands	AD	Years
10	Friend	50	F	Nursing	Theoretical	Netherlands	AD	Two years
11	Daughter	58	F	Nursing	Theoretical	Netherlands	AD	Years
12	Spouse	72	F	Theater production	Theoretical	Netherlands	AD	Two years
13	Son	40	M	Car repair/maintenance	Practical	Turkey	AD	Weeks
14	Daughter	46	F	Administration	Practical	Morocco (*Darija*)^c^	VaD	Ten years
15	Spouse	59	M	Sales	Theoretical	Netherlands	FTD	Years
16	Daughter	46	F	Stay-at-home parent	Practical	Suriname	AD	Years
17	Spouse	64	F	Education	Theoretical	Netherlands	AD	Weeks
18	Son	44	M	Car repair/maintenance	Practical	Turkey	AD	Weeks
19	Daughter	49	F	Nursing	Theoretical	Morocco (*Darija*)^c^	AD	Weeks
20	Daughter	44	F	Stay-at-home parent	Practical	Morocco (*Tamazight*)^d^	AD	Years

^a^M = Male, F = Female.

^b^Practical education emphasizes the acquisition of knowledge through hands-on, practical experience. Theoretical education (also referred to as academic education) focuses on the acquisition of knowledge, principles, concepts, and theories within a particular subject area.

^c^Darija, also known as Moroccan Arabic, is a language spoken by a subgroup of people in Morocco and may provide an indicator of the caregiver’s cultural background.

^d^Tamazight is a language family spoken by the Amazigh people of Morocco and may provide an indicator of the caregiver’s cultural background.

^e^PPA = Primary Progressive Aphasia, FTD = Frontotemporal dementia, AD = Alzheimer’s dementia, VaD = Vascular dementia.

## Ethical considerations

All participants provided written consent prior to the interviews. In each case, the informed consent form was either distributed via email beforehand or presented in person prior to the interview, at which time participants signed the document. After the study was completed, the audio recordings were destroyed. Only the transcripts are retained for scientific purposes as noted and in accordance with the outline in the informed consent form. The study was approved by the Institutional Review Board of the Erasmus Medical Center (MEC-2018-1568).

## Results

In this paper, we will present the results of the study by structuring them according to the following four themes identified in the study:

1.) Motivators to providing care
2.) Cultural perspectives on caregiving
3.) The role of spirituality and religion in caregiving
4.) Optimizing the care context for the caregiver

### Theme 1: Culturally shared motivators to providing care

#### A shared history

A variety of culturally shared reasons were reported for providing care for the person with dementia. A predominant motivator for informal caregivers was having an especially close relationship with the person with dementia. In those cases, providing care was perceived as self-evidently stemming from a shared history:

‘It’s been good for 48 years, and it will be good for another 48 if it continues like this. I know exactly how she thinks. I know exactly what she is like, her behavior. I understand her. I know how to interact with her.’ (Caregiver 5, Dutch heritage)

The shared history provided the caregiver with knowledge about the person with dementia and what the person with dementia would consider good care. The caregiver explained that this shared history enabled them to facilitate the best care based on all the details and intricacies they learned about the person with dementia throughout their lives. Some caregivers highlighted this as an important reason to provide care, as the person with dementia often could no longer advocate for their own wishes and needs due to the severity of the disease.

‘She is my mother, and I don’t know if there is anyone that would know her like I do. Especially now that my father has passed away. I know the soap that she likes to use when showering. (…). We also eat halal and I am not sure if others that don’t have our way of living would understand her.’ (Caregiver 13, Turkish heritage)

#### Reciprocity

Reciprocity was another cross-cultural reason for caregivers to provide care. Nearly all participants mentioned that they have in the past received care in some form from the person with dementia. For married couples, this was often considered in the light of the pledge made to one another:

‘We are married. Simple as that. You are there for each other in good and bad times. As long as it is possible for him to live at home, I will do my best to provide the best possible care.’ (Caregiver 12, Dutch heritage)

Adult child-parent care relationships were defined more often by how parents previously cared for the child, with children considering it ‘their turn now’ to provide care:

‘She has always given me a lot of love and care. I cannot leave her without any care. She is my mother.’ (Caregiver 14, Moroccan heritage)

Interestingly, some of the caregivers in child-parent care relationships emphasized the special bond they had with their parents in comparison with the relationship their siblings had with their parent. Some participants emphasized the amount of emotional support they had received from their parent during challenging times and how that strengthened their relationship:

‘She cared for me emotionally when one of my kids was in the hospital for a longer period of time. That was maybe one of the last times that she was very aware and caring towards someone before the dementia became noticeable. I had to go to the emergency room with my child and she really motivated me to stay strong for her. To make sure to be consistent with my prayers and that actually helped. I can never repay her for all the care and support that she provided during that difficult time.’ (Caregiver 13, Turkish heritage)

Many caregivers considered the element of not being able to (ever) ‘repay’ the person with dementia for the care they provided regardless of the type of care relationship.

Although reciprocity can be an important driver to provide care in families, other motives may come into play as well. One of the caregivers included in this study provided care for her father-in-law, who had always lived in China until he developed dementia. During the interview it emerged that the caregiver had had a complex relationship with her father-in-law in the past. When asking her about her motives to provide care, she stressed their family ties:

‘It is human nature. Without my help he can’t do anything. He doesn’t speak Dutch and because of the dementia his Chinese isn’t good either. He can’t explain things very well. He just needs somebody around and I do my best. (…) He is the grandfather of my son.’ (Caregiver 3, Chinese heritage)

### Theme 2: Cultural perspectives on caregiving

Caregivers of native Dutch patients—who might be presumed to be on the individualistic side of the individualistic-collectivistic continuum—more commonly reported considering care options outside of the home setting, such as adult day care services (in Dutch: “*dagbesteding*”) for the person with dementia:

‘There are still some things he can do. (…) To give myself a break I have searched for some forms of adult day care so I can have a few hours to myself. (…) I have also considered a long-term care facility and googled it. Just in case, so I know what the possibilities are.’ (Caregiver 4, Dutch heritage)

‘Look, when the time comes, I believe we will need to carefully consider what the right balance is considering our lifestyle. [We will need to evaluate] what care I can provide, and what care I cannot, and [consider] how I can maintain my own freedom in my own home while my husband becomes dependent on assistance. (…) I think considering care options outside the home can be a solution for that.’ (Caregiver 1, Dutch heritage)

These quotes show that caregivers of native Dutch patients were actively considering the use of specialized services, both now and in the near future. These considerations were driven by their desire to take a break from caregiving at times, but also to maintain some degree of autonomy over their own lives. They viewed care options outside the home as potential solutions to temporarily alleviate the caregiver burden they were experiencing.

In contrast, the caregivers of the patients from presumably more collectivist cultures did not spontaneously mention long-term care facilities or adult day care, but rather highlighted the number of individuals available to provide care in their personal network:

‘We are with 5 kids. I don’t think it is necessary to look into long-term care facilities. (…) We can do it ourselves.’ (Caregiver 14, Moroccan heritage)

This quote illustrates that the caregiver is prioritizing the quantity of available caregivers within the family over the potential benefits of professional care in terms of quality and relief. As mentioned earlier, she highlighted the importance of reciprocity in the care relationship for her mother, and it is likely that she expects this same reciprocity towards her mother from the other family members. This might be an explanation for why long-term care facilities or adult day care are not being considered. The culturally diverse caregivers also highlighted their commitment and willingness to persevere in the face of difficulties:

‘When I relate it to caring for my mother, I just know what needs to be done (…). Sometimes these are less pleasant things, but what can I do? It is a commitment I made. You already know at the outset what kind of process it can become.’ (Caregiver 13, Turkish heritage)

‘It is important to care for your parents. For how long will she [her mother] still be around? (…) I can persevere a little longer, so that at least she receives care from someone close to her.’ (Caregiver 19, Moroccan heritage)

### Theme 3: the role of spirituality and religion in caregiving

Some of the caregivers mentioned that spirituality and religion aided in creating a calm and stable state of mind throughout the caregiving experience. This was a common sentiment particularly among caregivers with a migration background. It helped them see the bigger picture:

‘You have more understanding for the situation of the other. (…) If you are very religious you will realize that He [Allah – God] will give you the ability to stay patient and do the job. He will not give you something that you can’t handle. That is a comforting thought.’ (Caregiver 14, Moroccan heritage)

Although religion was recognized as a way to cope with difficult situations, it was not necessarily identified as a reason to provide care. One of the participants who considered himself to be a religious person (i.e. Muslim) mentioned that he did not attribute providing care for his mother to his religion. Instead, he stressed his intrinsic motivation to provide care:

‘I am a Muslim. It is important to care for your parents because they are held in high regard. Well, I find it difficult to say that I only provide care for my mother because of my religion. That really isn’t just the reason for doing it. I really am willing to do it [provide care] and I do it in an attentive way. (…) I am just really motivated to do this for her.’ (Caregiver 13, Turkish heritage)

One caregiver viewed spirituality as a way of living in the present moment and applied similar reasons to their approach to caregiving:

‘I would say that I am spiritual to some extent. (…) I let everything wash over me for some reason. You can keep overthinking the situation as it is right now, but essentially those thoughts will change nothing. (…) So I live in the moment.’ (Caregiver 4, Dutch heritage)

Some caregivers did not identify with a specific form of religion or spirituality – this was common among caregivers without a migration background – but mentioned adhering to a personal philosophy of life:

‘It is more a way of life. The things you do for others should have a positive effect on them. Treat others the way you want to be treated. (…) Or maybe we should care for others in the same way you would like to be cared for yourself. That is the essence.’ (Caregiver 10, Dutch heritage)

This caregiver further elaborated on their view by stating the following:

‘Even if she will not remember a thing and will start cussing me out, I will always remain her friend and help her with whatever she needs. (…) My mother-in-law had a friend who also had dementia and when I once asked her why she doesn’t visit, she just said, without shame, that she [her friend] had nothing left to offer. (…) I would be terrified if I would live life that way.’ (Caregiver 10, Dutch heritage)

Even though some caregivers may not formally consider spirituality or religion a core element in their lives, they may still adhere to a certain philosophy on life in providing care.

### Theme 4: Optimizing the care context for the caregiver (facilitators)

#### The family environment

Caregivers reported a variety of facilitators that enabled them to provide care for the person with dementia. One facilitator that was mentioned often by adult children (in this study, mostly caregivers with a migration background) involved the provision of assistance from the caregiver’s spouse (or children), contributing to the smoother operation of their own family life:

‘My sisters both have daughters who can help out if there would be an important appointment. (…) I have also asked my daughters to stay with my mother when I needed to go to a family party.’ (Caregiver 14, Moroccan heritage)

‘I have a fantastic partner. Because of him all of this is possible. (…) If my husband would not be so understanding, we would not be able to provide care in the way that we do. Especially, with me also sleeping some nights of the week with my mom at her home. (…) He keeps the family running while I provide care for my mother.’ (Caregiver 14, Moroccan heritage)

When asked directly how a caregiver’s family life impacts the level of caregiver burden, caregivers 13, 14, and 19 mentioned that knowing that everything is running smoothly at home would be ‘*one less thing*’ that they would need to worry about.

#### Structure and routine to prevent restlessness and confusion

Caregivers of patients with a recent diagnosis of dementia across different cultural backgrounds reported difficulties coping with the unpredictability of the person with dementia:

‘I don’t know what goes on in her head sometimes. It is very messy up there which makes her very unpredictable. (…) Just recently, there was a moment that she forgot where we live. She ended up in a completely different street from the one we live in. (…) At those times I really ask myself: how [ can she think like that]?’ (Caregiver 6, Dutch heritage)

The caregivers with a longer experience in providing care for the person with dementia would also highlight the patient’s unpredictable behavior. Respondent 19 explained how they coped in the following way:

‘In the first few weeks my mother would be very restless for no apparent reason. She ate and bathed, but still, she remained restless. I could not really figure out why she would be restless because she didn’t give me a coherent answer when I would ask what she wanted. (…) Later, by chance, we found out that when we moved her favorite rug she became restless. The same with changing up her favorite hijab [headscarf]. (…) At the time I didn’t realize that she would attach so much importance to that.’ (Caregiver 19, Moroccan heritage)

Some caregivers also pointed out the importance of having a good structure/schedule in place for the caregiving tasks and additional activities for the person with dementia. This would help them to optimize the care for the person with dementia and also make the caregiving experience more manageable:

‘Our week has a clear schedule. I spend a full day with her on Thursdays. For the rest of the week, I have a clear overview of when and for how long someone is with her. That could be either her receiving more care at home, as well as her going to an adult day care service. (…) Fridays are normally the days I have to myself.’ (Caregiver 11, Dutch heritage)

### Summary of the main findings and recommendations

Several of the quotes provided by the caregivers in this study provide an insight into ways health care professionals might tailor their services to suit the diverse needs of these caregivers. Table 2 provides an overview of our main findings in the column on the left, followed by our recommendations for care professionals for clinical practice on the right.

**Table 2.** Key findings and recommendations for care professionals.

Key finding	Recommendations for care professionals
The decision to provide care often stems from **a deep-rooted, lifelong, and unique bond** between the caregiver and the person with dementia	**Ask caregivers about their prior relationship with the person with dementia** and how they grew into the caregiving role
Caregivers may worry that professional caregivers do not know the person with dementia well enough to tailor to their needs and wishes. It may be **difficult to give up the task of voicing the patient’s needs and wishes**	**Inquire about any worries a caregiver has about someone else** (in the family or outside of the family) **providing care to the person with dementia.** What aspects would they be concerned about? How might the caregiver remain involved in representing the patient’s needs and wishes?
**Religion** is often considered as a source of strength in continuing to provide care, but **may not be perceived as a motivator to providing care**	**Be mindful about the different perspectives on providing care and do not make any assumptions about the role religion plays in providing care.** Perhaps ask caregivers whether they have sufficient time to themselves to engage in activities that improve their own well-being, which may include spending time on religion
Caregivers who are not religious may have **important personal philosophies on life** that drive their decision to provide care	**Inquire about perceptions on providing care.** Examine whether a caregiver has a specific view on life that motivates them to provide care. This may help start conversations about the meaning of good care to the person with dementia and potential aid from the social network or professional care in dementia caregiving
In some cultures, **caregivers may look mainly towards the (extended) family to provide care**. They may be hesitant to use professional care and/or lack information on, or trust in, the services that are available	**Discuss what care is currently being provided by which person in the care network around the person with dementia.** Discuss the type of care that may be needed in the future for the person with dementia with increasing dementia severity
	**Inquire how the caregiver perceives care tasks in light of this increasing dementia severity.** Try to be helpful in coming up with creative solutions. Let caregivers know that you will give them more information about the available professional services so that they are aware of the options should they want to consider them
Adult child caregivers highlighted the importance of their own **family life running smoothly** and the **support** they receive **from their own spouse or children**	**Inquire about support within, and well-being of, the adult child caregiver’s own family** (spouse/children)
Through experience, caregivers identified patients’ **need for structure and routine** to prevent restlessness and confusion	**Educate caregivers about patients’ need for structure and routine.** Provide everyday examples showing how patients may have difficulty with seemingly minor changes to the life they know and understand

## Discussion

This study explored the variation in motivators and facilitators for providing dementia care in a home setting reported by informal caregivers with diverse cultural backgrounds (Dutch, Belgian, Turkish, Moroccan, Surinamese, and Chinese heritage). Four main topics emerged from the qualitative interviews: culturally shared motivators to providing care, culture specific perspectives on caregiving, diverse perspectives on the role of religion and personal philosophies, and ways to optimize the care context.

## Motivators for providing care

The main motivator that informal caregivers mentioned was the close relationship they had developed with the person with dementia over time and their unique knowledge of the other. Providing care was also perceived from the perspective of reciprocity. This shared history was often seen as the basis of the reciprocity and generalized to some extent across the different care relationships and across cultures. Caregivers specifically stressed how they had intricate knowledge about the person with dementia and how this contributed to optimal care.

Caregivers highlighted their role in communicating the needs and wishes of the person with dementia. Our study echoes findings from Belgium, where caregivers reported that older Moroccan individuals with dementia were not able to voice their needs to healthcare professionals, resulting in worries about entrusting older Moroccans to the care of healthcare professionals (Berdai Chaouni & De Donder, 2019). This trend may also illustrate a key difference in caregiving approaches, where informal caregivers—with their closer connection to the individual with dementia—are better attuned to their needs, whereas professional caregivers may struggle to recognize these needs due to the absence of that unique bond. As recommended by Alzheimer Europe (2020), professional caregivers should actively engage informal caregivers in the care process, ensuring it is tailored to the needs and preferences of the person with dementia. In particular, our study highlights the importance of incorporating discussions about how family members can continue to represent the needs and wishes of the person with dementia, while also addressing the specific concerns caregivers may have in various areas of care. Berdai Chaouni and De Donder (2019) show that each caregiver may serve a different role, such as the ‘coordinator’ of care, the ‘main nurse’ providing day-to-day care, the ‘assistant’ who supports the main nurse, and the legal representative fulfilling all administrative responsibilities and serves as a (legal) guardian. Such knowledge on how care is organized within the family may be relevant for care professionals to identify how best to support the system of informal caregivers.

## Reciprocity: A double-edged sword

Our study highlighted the unique relationships between the person with dementia and their caregivers. Some adult child caregivers stressed that they could *never* repay the person with dementia for the care they provided to them when they were younger. This theme of reciprocity may therefore present as a double-edged sword, motivating caregivers but also potentially resulting in caregivers providing care beyond their personal limits, which could in turn result in emotional and physical exhaustion. Such exhaustion is a common outcome of caregiving, particularly in culturally diverse populations (Ahmad et al., 2020; Berdai Chaouni & De Donder, 2019; Franzen et al., 2021), although also evident among Dutch caregivers (Franzen et al., 2021; Van Der Lee et al., 2017)—suggesting that the pressures of caregiving transcend cultural boundaries. Thus, while the theme of reciprocity may drive caregivers’ dedication, it can also contribute to burnout and exhaustion when caregivers exceed their own limits in an effort to repay their loved ones.

## The complexity of caregiving relationships

Although reciprocity and a unique relationship were often mentioned, motives to provide care may not always stem from an intimate and loving connection that the caregiver and person with dementia share or once shared. Healthcare professionals should consider the multifaceted family dynamics which have led to the caregiver taking up the responsibility to provide care for the person with dementia. It is often a sequence of events that willingly or unwillingly led to a caregiver taking up their role (Schulz & Eden, 2016). It may help to ask caregivers how they came to be the (main) caregiver and what their prior bond with the person with dementia was like. Previous research in diverse populations has highlighted moral framing rules that may lead caregivers to provide care (Ahmad et al., 2020). Future research might investigate whether caregivers who provide care out of feelings of (filial) duty experience caregiver burden differently than caregivers who underscore their unique bond with the person with dementia.

In this study, caregivers who were likely from collectivistic cultures were less inclined to (spontaneously) mention professional care/assistance when discussing care for the person with dementia. The question arises whether this stems from a reluctance towards professional help (Phillipson et al., 2014) or from a lack of knowledge of, or perhaps limited trust in (the quality of), professional care services (Duran-Kirac et al., 2022; Jensen & Inker, 2015). Another explanation could be that caregivers may not always know much about dementia (Alzheimer Europe, 2020; Berdai Chaouni & De Donder, 2019), how dementia progresses, and which care challenges they may encounter in the future. Alternatively, families may make use of other practices, such as 24 hour rotational care, as opposed to using formal care, or they may perceive providing care as a familial responsibility (Nielsen, Waldemar, & Nielsen, 2021). Formal care may also be perceived as inferior in quality to family care (Van Wezel et al., 2016).

## The role of religion and spirituality in caregiving

A number of caregivers, most of them with a migration background, expressed a need for spirituality and religion to continue to provide care. Various studies have considered religion or spirituality as an important aspect in the caregiving experience (Rathier et al., 2015; Stuckey, 2003). Some studies even identified religion as a main or predominant reason to provide care, with one interviewee stating, “*If it’s not for Allah, then you wouldn’t do it*” (Ahmad et al., 2020). In contrast, when we specifically inquired about the role of religion in our study, we found that religion was often described as a source of strength, making it easier to persevere in providing care, rather than being a main reason for providing care. Raising this question often resulted in caregivers mainly elaborating on their personal, intrinsic motivation. Based on our findings, caregiving should not be perceived as universally driven by religious beliefs across all religious caregivers. Furthermore, religion and spirituality in our study did not always cover all the ways in which people give meaning to their life and the caregiving experience; some may adhere to specific life philosophies without identifying with any particular religion.

## Facilitators to care

Last, caregivers highlighted facilitators to care, which included support received from their own spouse or children, as well as adhering to routines and structure to prevent the patient becoming restless, confused, or showing unpredictable behavior. This was especially relevant for adult children (who, in this study, often had a migration background) providing care for their parents. Care professionals may support caregivers by providing education about the behavioral and psychological symptoms of dementia, other dementia related symptoms, and the dementia trajectory. Furthermore, interventions are slowly being developed to assist culturally diverse families in providing care; for example, a pilot study in Denmark revealed that caregivers appreciated having someone to turn to for questions on “smaller everyday issues” in providing care, as well as someone to help with “coordination of care services” (Nielsen, Waldemar, & Nielsen, 2021).

## Strengths and limitations

The findings of this qualitative study contribute to the literature by offering insights into motivators and facilitators to providing care. Strengths of this study include the inclusion of caregivers with different care relationships (i.e. spouse, adult-child, and friend), as well as the different dementia diagnoses and countries of origin of the patients (including caregivers of both native Dutch patients and patients with a migration background). Furthermore, our study included a number of male caregivers, a group that was not represented in previous studies (e.g. in Ahmad et al., 2020; Van Wezel et al., 2016). Another strength was that interviews were conducted by a bilingual and bicultural researcher, while coding was conducted by researchers with different cultural backgrounds. A limitation of this study is that we conducted all interviews in Dutch, which may have influenced the way caregivers expressed their experiences. Furthermore, a small subset of the interviews was conducted by phone or through videoconferencing, which could potentially have impacted the dynamics during the interview. In some cases, using such methods was necessary, because of logistical barriers or the caregiver not being able to host the researcher at home without the person with dementia present. This would hinder talking freely about their experiences. A third limitation is that the adult-child caregivers often had a migration background, while the caregivers of patients born in the Netherlands were spouses. This makes it difficult to disentangle the effects of migration background/culture from the relationship type and the age of the caregiver. Finally, we examined the effects of culture by including participants with several different cultural backgrounds in our study. As the sample was selected to be as diverse as possible to uncover as many different perspectives as possible, we included only small numbers of participants per cultural group. The disadvantage of this strategy is that some culture-specific aspects particular to one cultural group may have been missed. A follow-up study in specific, homogenous cultural groups may be needed to examine such culture-specific factors in extensive detail.

## Conclusions and recommendations

In summary, the number of diverse older adults with dementia will increase over the next decade; current policies directed towards aging in place can only succeed if the experiences of culturally diverse informal caregivers are taken into account, lifting barriers, but also promoting facilitators to provide care. This study highlights four relevant themes in this context: 1) motivators to provide care, 2) cultural perspectives on caregiving, 3) the role of spirituality and religion in caregiving and 4) optimizing the care context. Several recommendations within the four themes were made that can help healthcare professionals gain a better understanding of the broader care system in which informal caregivers operate. This will hopefully lead to better support of the culturally diverse, informal caregivers devoting so much of their time and energy to caring for their loved one with dementia.

## Supplemental Material

Supplemental Material - Cultural variability in dementia caregiver motivations: Unraveling unique and common drivers Supplemental Material for Cultural variability in dementia caregiver motivations: Unraveling unique and common drivers by Najoua Lazaar, Floor Flurij, Rozemarijn L van Bruchem-Visser, Janne M Papma, and Sanne Franzen in Dementia
