# Supplementary material for: Cultural variability in dementia caregiver motivations: Unraveling unique and common drivers
Source: Dementia (London). 2025 Apr 14;24(6):1115–33. doi: 10.1177/14713012251327461 (PMC12276403; doi:10.1177/14713012251327461)
Supplement: Supplemental Material - Cultural variability in dementia caregiver motivations: Unraveling unique and common drivers [file sj-pdf-1-dem-10.1177_14713012251327461.pdf]

Interview guide: Cultural Variability in Dementia Caregiver Motivations: Unraveling Unique and Common Drivers

Opening remarks

- *Brief introduction and explanation of the research/interview*

This research aims to explore various factors that contribute to sustaining care for people with dementia in the home setting. We seek to understand what daily caregiving looks like, the motivations driving caregivers to provide care, and how different aspects may influence the ability to continue to provide care over time.

- *Inquire if there are any questions regarding the consent form that was previously sent.*
- *Clarify confidentiality and how the information will be used.*
- *Answer any additional questions.*
- *Sign consent form.*

<u>What does caregiving look like for you?</u>

- Can you describe what a typical day looks like when you provide care for a person with dementia?
- What are the key activities you assist with?
- In which areas does caregiving go well, and why? Could you describe a specific situation?

<u>Caregiving and strength</u>

- Who and/or what helps you to provide care for your loved one effectively and sustainably over time?
- What is your primary motivation for providing care?
- How do you handle situations in which others offer to assist with caregiving for the person with dementia?
- What, in your opinion, constitutes good care for your loved one?
- Why did you[1] choose this way of caregiving?
- Have there been things in your life that you have had to adjust to provide care for your loved one? Why do you do that?
- At which moments do you see that the care you provide is making a positive impact?

<u>Faith/religion/spirituality: helping the vulnerable and reciprocity</u>

- Does faith/religion/spirituality play a role in your experience as a caregiver? If so, in what way?
- How do traditions influence your experience as a caregiver? If applicable, how do they manifest in your caregiving approach?
- Have there been moments when you have received care from the person to whom you now provide care? Can you elaborate on that?

<u>Resilience: the ability to cope with change and difficulties</u>

- What effect has this caregiving experience had on you?
- Can you describe a moment or situation when caregiving was particularly difficult, and how you were ultimately able to persevere?
- Do you receive support during the caregiving process? If so, in what ways?
- How do you manage to keep going despite the challenges?

---

[1] A Dutch singular form of the word you ("jij") and a plural form ("jullie") are used in the original guide.

<u>Adaptability: adjusting and finding a way in a new situation</u>

- How did you arrive at the point where you are now, providing care in the way you do?
- What skills, personality traits, or characteristics do you possess that are important for caregiving to your loved one?
- What impact has this change had on you?

<u>Closing remarks</u>

- Is there anything else you would like to share about your experience as a caregiver?
- Do you have any final thoughts or reflections on how caregiving has shaped your life?

Literature

Ahmad, M., van den Broeke, J., Saharso, S., & Tonkens, E. (2020). Persons With a Migration Background Caring for a Family Member With Dementia: Challenges to Shared Care. *Gerontologist*, *60*(2), 340-349. https://doi.org/10.1093/geront/gnz161

Alzheimer's Association. (2021). 2021 Alzheimer's disease facts and figures. *Alzheimer's & Dementia: The Journal of the Alzheimer's Association*, 17(3), 321-552. https://doi.org/10.1002/alz.12328

Babbie, E.R. (2020). *The Practice of Social Research* (13 ed., Vol. 1). Cengage Learning.

Creswell, J.W., & Poth, C.N. (2013). *Qualitative inquiry and research design: choosing among five approaches*. Sage Publications.

Nielsen, T.R., Nielsen, D.S., & Waldemar, G. (2021). Barriers in access to dementia care in minority ethnic groups in Denmark: a qualitative study. *Aging & Mental Health*, *25*(8), 1424-1432. https://doi.org/ https://doi.org/10.1080/13607863.2020.1787336

Nielsen, T.R., Waldemar, G., & Nielsen, D.S. (2021). Rotational care practices in minority ethnic families managing dementia: A qualitative study. *Dementia: The International Journal of Social Research and Practice*, *20*(3), 884-898. https://doi.org/ https://doi.org/10.1177/1471301220914751

Van Wezel, N., Francke, A.L., Kayan-Acun, E., Deville, W.L.J.M., Van Grondelle, N.J., & Blom, M.M. (2016). Family care for immigrants with dementia: The perspectives of female family carers living in The Netherlands. *Dementia: The International Journal of Social Research and Practice*, *15*(1), 69-84. https://doi.org/https://doi.org/10.1177/1471301213517703
